# Acceptability and Feasibility of Callascope Self-Imaging of the Cervix among Women in Ventanilla, Peru: A Mixed Methods Pilot Study

**Published:** 2021-04-02

**Authors:** Mary E Dotson, Elizabeth Cuenca, Marlee Krieger, Nirmala Ramanujam, Patricia Garcia

**Affiliations:** 1 Department of Biomedical Engineering, Duke University, Durham, North Carolina, United States; 2 Department of Global Women’s Health Technologies, Duke University, Durham, North Carolina, United States; 3 Department of Duke Global Health, Duke University, Durham, North Carolina, United States; 4 Department of Public Health and Administration, Cayetano Heredia University (UPCH), Lima, Peru

**Keywords:** Speculum-free imaging of cervix, Primary prevention, Acceptability and feasibility

## Abstract

**Background:**

Barriers that prevent adherence to cervical cancer screening programs in low-income communities include, among others, fear of and discomfort associated with the speculum during pelvic exams and difficulties in accessing health facilities. To address these barriers, a low-cost medical device, the Callascope was developed for self-imaging of the cervix. The device has a 2 MP camera connected to a smartphone and a disposable inserter with an asymmetrical tip to replace the speculum. A pilot study was performed to 1) evaluate the feasibility, acceptability, and comfort of women imaging their own cervix with the Callascope, 2) collect information to improve the design of the Callascope prototype, and 3) identify factors related with the ease of use, discomfort of the self-exam technique and quality of images.

**Methods:**

The pilot study included women (n=15) who were either current or former community-health volunteers from Ventanilla, Peru with the HOPE program. Participants completed a pre-exam survey to assess demographics and establish reproductive medical history. Each participant was provided with a self-exam kit. They were asked to perform a self-exam and take pictures of their cervix with the Callascope at home at their convenience. They submitted an audio reflection immediately post-examination via WhatsApp. A post-insertion survey to assess user experience and a focus group discussion were performed 72 hours post-insertion.

**Conclusions:**

The Callascope self-imaging of the cervix was reported to be more acceptable and comfortable than the traditional speculum-based gynecologic exam. All participants indicated that they would use the device again and recommend to a friend. Recommendations to improve the prototype design were identified: lengthen handle, overlay asymmetrical tip with softer silicon, and optimize manual focus mechanism. The Body Mass Index (BMI) was found to be a factor associated to the ease of device use and comfort.

## Introduction

In 2018, incidence of cervical cancer was 570,000 cases globally with 311,000 deaths from the disease [1,2]. Cervical cancer is the second most common cancer in women worldwide and the first in several Low And Middle Income Countries (LMICs), like Peru, where it is also the leading cause of cancer deaths among women [3,4]. More than 80% of incident cases and 85% of all mortality occurs in LMICs, mainly in Latin America, sub-Saharan Africa, and the Indian subcontinent [5,6]. In contrast, High Income Countries (HICs) have had tremendous success in decreasing the burden of disease due to effective screening programs and infrastructure that supports follow-up and treatment [7,8]. Several socioeconomic risk factors prevalent in LMICs are associated with this disparity, including national poverty rates and health expenditure per capita, rates of urbanization and literacy, and gender inequality [9,10]. Overall, the disproportionate burden of cervical cancer is due to the presence or absence of well-organized and effective screening programs with linkages to follow-up and treatment [11,12]. Just recently, on November 17th, 2020, the WHO launched their Global Strategy to accelerate the elimination of cervical cancer following a call to action announced by the Director-General in 2018 [13-15].

In contrast to many cancers where multiple determinants can contribute to carcinogenesis, cervical cancer has one central causal agent, the Human Papillomavirus (HPV) infection. HPV is mainly sexually transmitted and can be prevented through a vaccine which is effective and safe. However, vaccine implementation has not translated into universal, equitable access due to high costs, poor delivery infrastructure, and general lack of community awareness in both LMICs and HICs [16]. On the other hand, the carcinogenesis process caused by the expression of viral oncogenic proteins E6 and E7 induced by the integration of the human papillomavirus into the host genome, results in slow progression of the disease and a long-latency period which offers opportunity for prevention and early detection [17,18]. Liquid-based cytology and the “pap” smear are gold standard screening tests used in HICs, involving the collection of cells from the cervical lining and identification of abnormal cell growth [19]. These tools have dramatically reduced incidence and mortality from the disease in developed countries with the PAP test alone being responsible for reducing incidence by 70% in the US between 1955 and the mid-1980s [19]. However, structural barriers make sustaining high-quality cytology-based programs difficult in low-resource settings [20-23]. In addition, several social barriers prevent women to access to cervical cancer screening programs-lack of information about the disease, fear and pain associated with the pelvic-exam and the use of the duck-bill speculum, as well as cultural and religious stigma associated with sexual and reproductive health [24,25].

Detecting the presence of HPV in the cervix is another option for screening, when paired with self-sampling this method can increase test coverage and is very sensitive to detect lesions. It is recommended in women 30 years and older and could be performed every 3 to 5 years [26-28]. Nevertheless, once a woman is positive on any of the screening tests, she will be required to undergo evaluation with a Colposcope, to inspect the cervix and decide treatment [29]. Colposcopes are expensive devices and are not found easily in most primary care centres, which forces women to travel to the few urban-cantered establishments that have the device and the personnel trained to use them. Consequently, colposcopy is inaccessible to the most vulnerable women living in LMICs, those who are at the highest risk of developing cervical cancer. Many women with apparently positive lesions do not complete their diagnosis or treatment due to fear to the examination or lack of service options. This problem encourages working on the creation of new cervical cancer screening models that include the implementation of new technologies.

**Figure 1:**
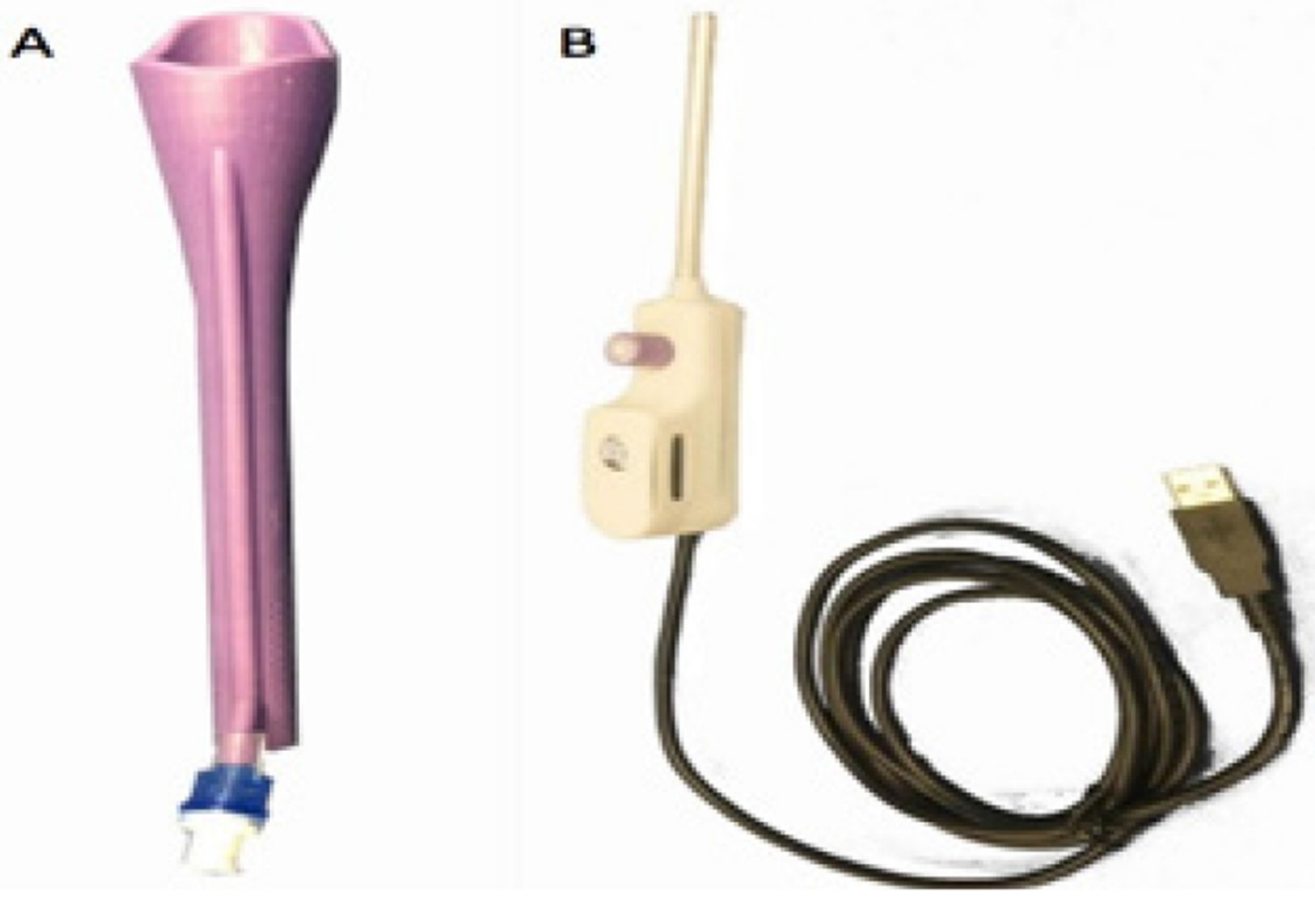
Callascope Inserter and 2 MP cameras - A) Callascope inserter B) 2 MP Camera. The slender 2 MP camera design (B) fits into a disposable Cala Lily shaped, asymmetrical inserter, which replaces the duckbill speculum (A). The camera and inserter include a handle which connects to a computing device (Android cell phone or laptop) with a software application that enables women to complete self-examination privately from a primary clinic facility or at-home.

**Figure 2:**
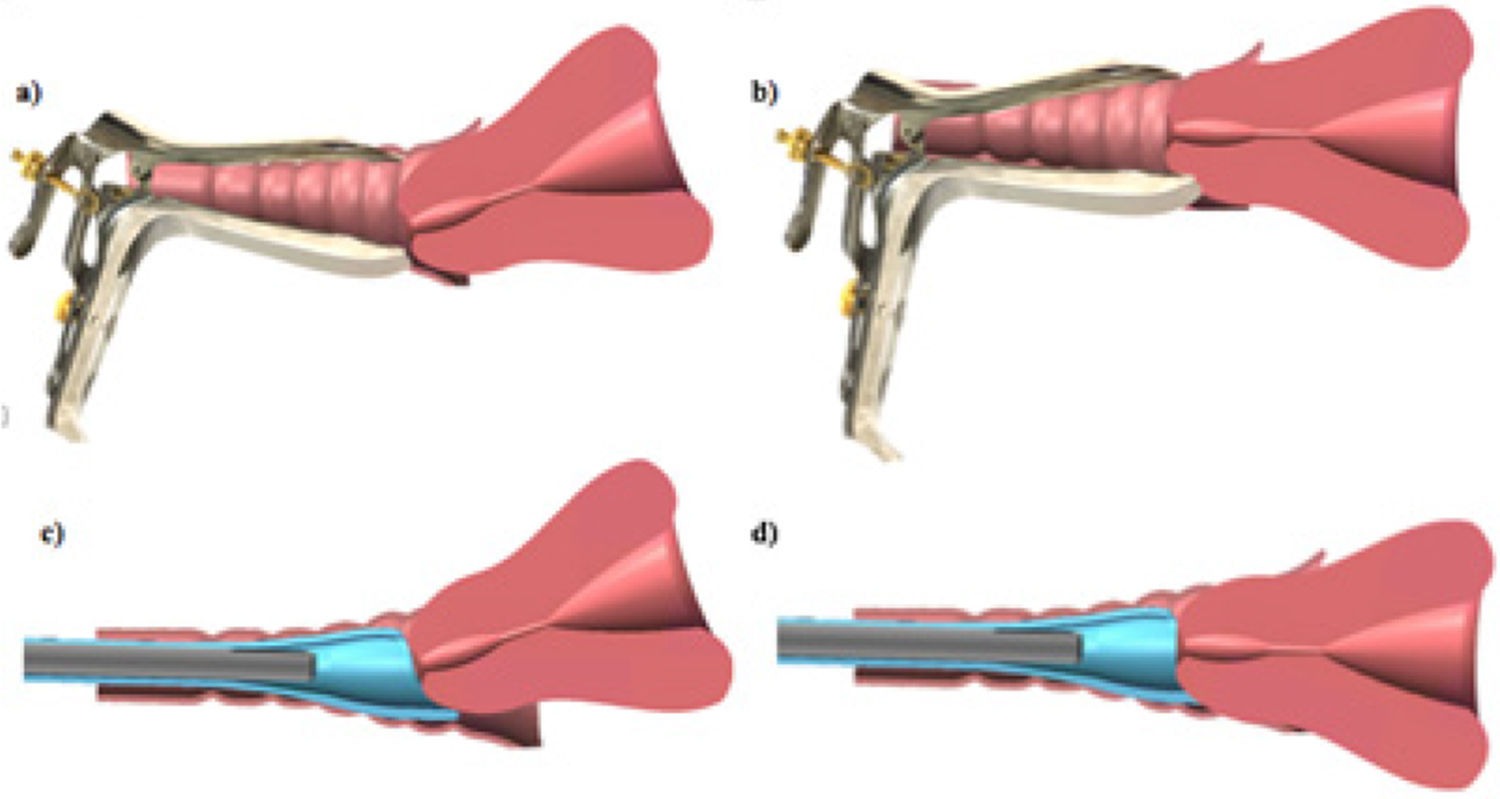
Traditional speculum versus callascope for autonomous self-imaging. a) Traditional speculum with tilted uterus, b) Traditional speculum centralizing the cervix for imaging, c) Callascope in the vaginal canal with tilted uterus, d) Callascope centralizing the cervix for imaging.

The Callascope is one of those new technologies. It is a portable, low-cost, easy to use device that allows for self-imaging of the cervix and lower reproductive anatomy [30,31]. It has the potential to address several of the aforementioned barriers to primary cervical cancer prevention. The slender 2 MP camera fits into a disposable Cala Lily shaped, asymmetrical inserter, which replaces the duckbill speculum. The camera and inserter include a handle which connects to a computing device (Android cell phone or laptop) with a software application that enables women to complete self-examination privately from a primary clinic facility or at home (Figure 1). The camera is placed into the inserter and then gently guided into the vaginal canal with lubricating jelly. The device is pushed into the canal until the cervix comes into view, and then the asymmetrical tip is rotated until the cervix is moved into focus (Figure 2). The woman can then take images with the computing device and save them within her medical record for review by a medical provider. It has been initially evaluated in Accra, Ghana and Durham, NC, USA [30] though all of these prior studies were performed in a clinical setting (hospital) under supervision by a nurse or physician.

In this article we report the results of a pilot study designed to evaluate the acceptability and feasibility of the use of the Callascope by Peruvian women to complete a self-exam to image their cervix at home, as well as to identify specific prototype design modifications to improve the device.

## Methods

### Study design and participants

We conducted a mixed-methods study involving Peruvian community women from Ventanilla, a low Socio-Economic Status (SES) district located in the desertic coast, geographically located east from the capital city of Peru, Lima. Eligible participants were women, 18 years of age or older, who participate in the HOPE program (HOPE Ladies) and had no history of hysterectomy, LEEP procedure, were not pregnant and were not having their menstrual cycle at the time of device use and self-examination. The HOPE program (https://hopeperuproject.org/project/) is a social enterprise promoting HPV self-sampling in Peru. HOPE promotes the participation of community women as agents of change through becoming community-health workers (HOPE ladies), the use of self-sampling with low-cost HPV DNA-molecular tests and linkage to a tracking system to ensure timely result delivery and further follow up. Due to novel concept of self-examination, participants who were already sensitized to reproductive health were chosen for this study. The sampling frame included 59 HOPE ladies working for the HOPE program in Ventanilla. All of them were invited to participate and from those eligible a list was developed of final participants in order of registration. Due to the limited number of prototypes available for the study, we anticipated 15 women maximum to enrol in the study.

### Study procedures

Potential participants were invited to a first meeting which happened at a central location in Ventanilla in March 2020. At entrance, eligibility criteria were reviewed by a member of the research team which also explained the procedures of the study in detail. If the HOPE lady agreed to participate they signed the informed consent. The meeting started by asking participants to complete an interviewer-administered pre-examination survey including questions about age, BMI, marital status, occupation, education, participation in the HOPE project, reproductive health history (e.g. number of vaginal births, experience with previous speculum exams), and initial perception of the callascope. Following the survey, participants underwent a group session performed by the two midwives from the project team. The session included a demonstration and walk-through of the self-exam with the Callascope and distribution of instructions. During training, participants were given a demo version of the Callascope to familiarize themselves with the size, shape, and color. At the end of the meeting each participant was provided with a self-exam kit including two-pages of written instructions, one Callascope, disposable Cala shaped lily inserters in two different sizes, small and large (Figure 1), an Android cell phone, charger, micro-USB adapter, lubricating jelly, feminine sanitation wipes, and anti-fog wipes for the camera lens. They were asked to perform a self-exam and take pictures of their cervix with the Callascope at home at their convenience before the debriefing meeting. Additionally, they were asked to record an audio reflection of their experience immediately post-examination and send it to the study staff via WhatsApp [32]. Audio-reflections were unstructured, and participants were asked to keep their recording at 10 minutes or less.

Participants returned for a follow-up visit 72 hours after enrolment to complete the post-exam survey, drop off their self-exam kit, and participate in the focus-group activity. The post-insertion survey included 15 questions regarding participant experience completing a Callascope-enabled self-examination. Participants rated the ease-of-use, comfort, and pain experienced using a Likert 5-point scaling system (agree to disagree) [33] and the Wong Baker FACES pain scale [34]. Upon completion of the post-insertion survey, participants convened in an audio-recorded focus group facilitated by the study coordinator (midwife). They were asked to summarize their overall experience with the procedure, to discuss any discomfort experienced during self-examination, to identify workflow challenges in completing the procedure at-home, and to provide feedback on the medical device design and components provided in the self-exam kit.

### Data analysis

#### Quantitative analysis:

Analysis of the pre-and post-survey was performed using R version 4.0 (R Foundation for Statistical Computing, Vienna, Austria). Descriptive statistics were used to summarize responses to both surveys. The Spearman rank sum correlation coefficient was applied to evaluate the association between two key independent variables-BMI, number of prior vaginal births-and dependent variables including-ease-of-use, discomfort, pain level, and image quality. The Rho and P-values were calculated to estimate both the direction and strength of association. Image quality was evaluated by an obstetrician, defining if it was a good or bad image on the following criteria: 1) captured image of cervix 2) clarity or focus, and 3) centring of cervical os with all four quadrants visible. Each category was given a rank between 0 and 2, which was aggregated into one numerical rank score for analysis. BMI was calculated using standard international guidelines of height and weight. Ease of use, discomfort, and pain level were all calculated on a Likert or Wong Baker scale and converted to a numerical rank scale.

#### Qualitative analysis:

The audio-reflections submitted by participants via WhatsApp and the audio recording of the focus group were both transcribed and de-identified by study personnel at a later date. Transcripts were analyzed in Spanish using a validated qualitative data analysis platform, NVivo v12. Direct content analysis was used to deductively evaluate the focus group and reflection data with predetermined codes obtained through observations and field notes taken by study personnel. Major themes were identified in alignment with the four categories of inquiry including, 1) overall experience, 2) comfort, 3) procedure workflow, and 4) device design and components. The study protocol and instruments were reviewed and approved by the Institutional Review Board at the Cayetano Heredia University (UPCH) in Lima, Peru (Ref. Ethics Committee 102926).

### Study design and participants

We conducted a mixed-methods study involving Peruvian community women from Ventanilla, a low Socio-Economic Status (SES) district located in the desertic coast, geographically located east from the capital city of Peru, Lima. Eligible participants were women, 18 years of age or older, who participate in the HOPE program (HOPE Ladies) and had no history of hysterectomy, LEEP procedure, were not pregnant and were not having their menstrual cycle at the time of device use and self-examination. The HOPE program (https://hopeperuproject.org/project/) is a social enterprise promoting HPV self-sampling in Peru. HOPE promotes the participation of community women as agents of change through becoming community-health workers (HOPE ladies), the use of self-sampling with low-cost HPV DNA-molecular tests and linkage to a tracking system to ensure timely result delivery and further follow up. Due to novel concept of self-examination, participants who were already sensitized to reproductive health were chosen for this study. The sampling frame included 59 HOPE ladies working for the HOPE program in Ventanilla. All of them were invited to participate and from those eligible a list was developed of final participants in order of registration. Due to the limited number of prototypes available for the study, we anticipated 15 women maximum to enrol in the study.

## Results

### Quantitative results

#### Post-exam survey:

Participant age ranged from 31-59 years of age; median age was 45 years old. Just over half (54%) had some post high-school education. Forty percent were identified as overweight (BMI over 25 and less than 30) and just over half (53%) were as obese (BMI>30). Eighty percent of participants reported their relational status to be married or cohabiting and sixty percent reported their occupation as a housewife. Just under half (47%) reported their highest education level as high school (secondary), and the majority reported some technical or university education (53%). Nearly half reported having worked with the HOPE project for 12 or more months (47%). The average number of vaginal deliveries was 2.1 (range 0 to 5), with one-third reporting 3 or more vaginal births. All participants self-reported that they had experienced at least one speculum-based pelvic exam in the past. The average number of previous speculum-based pelvic exams was 8.6 (range 2 to 20) with the majority (60%) reporting having had experienced between 6 and 10. Just under half (46.7%) reported their experience with speculum-based pelvic exams to be characterized by severe pain and discomfort. As part of the initial survey, two-thirds of participants indicated that they felt the Callascope self-examination would be hard to complete autonomously (Table 1).

#### Post-exam survey:

Ninety-three percent of participants reported that the provided self-exam instructions for use of the Callascope in their homes were easy or very easy to follow. Twenty percent (3/15) used only the small size disposable Cala shaped lily inserters, 53% used only the larger size and 27% tried both sizes. The reported time to complete the self-exam averaged approximately 4 minutes (median 2 min; range 1-20 min). Forty-seven percent of women (7/15) reported that it was easy or very easy to locate their cervix. Eighty percent (12/15) reported none or slight discomfort during use and the same percent reported no pain when moving the device for visualization of the cervix. All women reported the Callascope self-exam as more comfortable than the speculum-based pelvic exam and that the experience using the Callascope self-exam was better or much better than previous experiences with speculum-based pelvic exams. All participants indicated that they would complete a self-exam with the Callascope again and would recommend the device to friends or family (Table 2).

#### Cervical images:

The total number of images taken by each participant ranged from 15 to 75 photos; mean total photos taken per participant was 28.5. Forty percent (6/15) women took at least one photo considered of good quality. Of the nine women who did not adequately visualize their cervix five had a BMI greater than 30 (obese) and 7/9 reported having had 3 or more vaginal births. We found an association between BMI and the ease of use of Callascope (Rs=0.038) (higher BMI, harder to use) and between higher BMI and pain when manipulating the Callascope to visualize the cervix (p=0.021) (Table 3).

### Qualitative results

#### User experience:

Nine participants discussed feelings of fear and apprehension prior to completing the exam during the audio reflections and focus group. They attributed those fears to the device texture and/or concept of self-imaging their own cervix. One participant stating, “At first I was scared when I saw the device.” But it was also clear, that once they tried the Callascope, fears disappeared. Some women tried to complete the self-exam privately, but many others completed the exam with assistance or guidance from household members such as the husband or their close relatives living at home. Women highlighted the importance of following the instructions for the use of the Callascope and of having some distance support.

Most women indicated no discomfort during the self-examination. Some women specifically stated that the experience using the Callascope was much better (comfort, fear, etc.) than a speculum-based pelvic exam. All participants refer the use of the Callascope as a positive experience as shown by the following quotes: “This was a stunning experience for me because I got to know my inner body” “for me it has been a very pleasant experience, because it is the first time that I can see (this) part of my body, my organism through a camera.” Since all women reported they would recommend the use of the Callascope to other women or were willing to try it again, we asked them why. Most women indicated that experience had allowed them to learn more about their reproductive anatomy, others, highlighted the ease of use and comfort of the device and highlighted the novel, inquisitive, and empowering experience of self-examination itself (Table 4).

#### Challenges encountered during self-examination:

In the post use meeting, all participants were prompted to provide feedback on challenges experienced during the self-exam. The two primary issues that arose during the discussion: 1) fear and doubt related to unexpected “bodily fluids” in the vaginal canal (vaginal discharge); and 2) participant’s difficulties locating cervix and uncertainty around its correct identification. Some women complained that the “fluids” were not allowing having a good image, and others were concern if it was normal or abnormal and what was causing the discharge. Many of these women highlighted that they were unaware that they had discharge, leading to uncertainty about whether it was normal or an indication of poor hygiene or poor health. Women expressed their difficulties finding and correctly identifying their cervix with the device, “Later, at the moment of looking for the cervix, I honestly could not find it, so I got even more scared. Where is it? Am I not doing it right or what”? Other women had issues manipulating the device to obtain a good image, “It was a bit difficult I have turned (the device) many times” (Table 5).

#### Device Design Recommendations (DDR):

All participants were prompted to provide feedback on the design of the Callascope device during the focus group. Four design modifications were recommended by all fifteen women: 1) The material comprising the asymmetrical Cala lily-shaped tip for manipulation of the cervix should be softer than the rest of the inserter body. Participants stated that the current tip was “too rough and too hard,” evoking fear and doubt about using the device during training, with two participants reporting that they could feel the rough tip during the exam. One participant stated that “the contour material could be a little softer, which is the part that is going to take (touch) the cervix, right?” 2) The inserter handle should be lengthened for easer device manipulation and use. One woman stated “Well the suggestion that I would make is the shaft. Why? Because I am a little plump, it was a little difficult for me, so I think if it was a little bit longer because there are bigger people too, so that’s the suggestion.” 3) Inclusion of additional inserter sizes, as well as clear instructions explaining which size to use based on height and weight. One woman said, “I would say that maybe with the weight, with the size that we have, maybe some women can use the (Calla size) one, some women can use the (Calla size) two, so that maybe someday if we do this, right, go out to the field and ask (women) how many kilos do you weight and what size are you, then we would already know what things to give them or do.” 4) Remove manual focus and replace with an automatic focusing mechanism or fixed working distance. Many women stated that it was overwhelming to have to manipulate and rotate the device inside their vaginal canal while turning the small manual focus knob located outside of the body. “Well, for me, what bothered me the most has to focus; focusing so that the photo comes out sharp? If maybe it was in a fixed position, in which one would just click on the cell phone and your tone would smoothly come out, because that's what worried me the most, being able to turn, turn to find the best shot. The rest is ok, very good”.

## Discussion

Experts across disciplines agree that participation in cervical cancer screening programs is a critical factor for improving patient outcomes. However, compliance to screening guidelines remains an issue in LMICs for a variety of structural and social reasons including-fear and discomfort associated with the pelvic exam and speculum, as well as poor access to services [35,36]. HPV self-sampling bridge these barriers by offering women the option to initiate screening at home, reducing potential financial and logistical burdens to the patient, and allowing for a greater sense of privacy and autonomy. Several recent studies have shown that HPV self-testing strategies overwhelmingly increase participation and compliance with cervical cancer screening while actually motivating patients to undergo follow-up care [37-41]. However, after a positive HPV testing, the next step is to perform the visualization of the cervix which requires going to health center and having a pelvic examination with speculum performed by a specialized medical doctor. To our knowledge, this is the first study to explore the acceptability and feasibility of an autonomous self-examination procedure to visualize and image the cervix in a non-clinic setting, which could be an alternative to increase the compliance with cervical cancer screening. Study participants all completed the Callascope-enabled self-exam and indicated that the device was more comfortable than standard gynecological procedures that they would use the device for a self-exam again, and they would recommend the device use to friends and family. All participants reported a positive experience overall, relating this to its educational capacity and ability to give them control over a procedure they once knew nothing about. These results are similar to the acceptability findings from other self-testing procedures for HPV and HIV [42-47]. Women feel empowered to manage their own health.

### Recommendations for improving the callascope design:

While study results indicate the device was acceptable and all women successfully completed the self-exam procedure, participant’s encountered notable feasibility issues that should be addressed through modifications made to the engineering design before Callascope self-imaging can be implemented in broader applications. User feedback indicated some issues in locating the cervix during the self-exam. Participants reported that this was the manual focusing mechanism, the length of handle for device manipulation. These recommendations should be top priority for the engineering team, as they directly impact image quality, clinical utility, and user experience. A 2 to 5 MP color CMPS auto-focus imager or Autofocus (AF) optical system has been integrated into another provider-based cervix imaging device, the pocket Colposcope, with great success, suggesting it could integrating into the Callascope design to address the user feedback [48]. Additionally, Body Mass Index (BMI) was shown both qualitatively and quantitatively to be an important consideration for the self-exam technique. In the future, the self-exam kit should include a variety of inserter sizes with clear indications for use based on BMI and height. Modifying the Cala Lily tip with softer silicon is another design modification to improve patient comfort and mitigate pre-exam concerns. Furthermore, women identified as a challenge the presence of “vaginal fluids” which caused difficulties to visualization as well as fear during the procedure. This should be taken into consideration in the improvement of the device design, and the user instructions for self-examination should include information informing participations about vaginal discharge.

The incorporation of user-feedback during the device development process has been the focus of many publications [49-51] Both, the Association for the Advancement of Medical Instrumentation and the FDA have reported that this user-cantered approach is essential to assured medical device safety [52,53]. User involvement has been recognized as having other health related benefits including; improved patient safety, better health outcomes and compliance, and higher levels of patient satisfaction and uptake [54]. Some evidence suggests that the approach increases the likelihood that a device will be commercially successful downstream [55,56] Sandhu and colleagues at Berkeley’s Public Health Institute posited in a review article [57] that by analysing problems in their true context with people’s real experiences at the forefront, a human-cantered approach to device development can shift community medicine from the prescription of solutions according to our perception of needs in western institutions, to identifying and co-creating solutions that actually meet the challenge where the community is and not where we think they are as public health practitioners. A poignant example of what can happen if the end-user is not involved in the design is the FemSpec, a clear cylinder and inflatable tube with plastic air pockets, which was developed in 2005 by Femsuite to expand the vaginal walls without the need for a speculum [58]. The device was not successful, later removed from the market due to provider reluctance [58].

The Callascope device has been evaluated in other settings in Ghana and the US, and in contrast to this study, had comparable image quality to speculum-based examinations [30]. However, in both cohorts’ patients were able to complete the exam or training in a clinic with assistance from a trained practitioner. The pilot study in Ventanilla is the first to evaluate device feasibility in a fully autonomous non-clinical format. The differences in image quality observed between the three pilot cohorts reveals how context and implementation factors influence device usability, further demonstrating the importance of user involvement in the development process.

### Considerations for improving the self-exam kit and virtual training package:

Other critical user feedback can be addressed through revisions to the intervention training package and self-exam kit provided to users. The revised training package should provide a robust database of videos demonstrating the self-exam process, accompanied by a large sample of de-identified cervix images. An example could be the virtual educational program, the beautiful cervix project, which showcases photographs of changes in the cervix and cervical fluid throughout the cycle [59]. A package as such, could improve woman’s ability to locate cervix and mitigate anxieties related to vaginal discharge or cervical fluid and could be provided to Callascope users as part of the proprietary software application or as a separate free downloadable application for use after exam completion. Moreover, information about other causes as Sexually Transmitted Infections (STI) could be also provided. Such Digital Health Interventions (DHI) have been shown to improve public health tools and their capacity to improve healthcare delivery by increasing their effectiveness, efficiency, and safety [60], further they are increasingly being adopted to foster behavior change and reduce intervention costs [61]. A digital training strategy could reduce logistic expenditures, personnel and training costs, reducing the burden to health systems and creating space for telemedicine guided self-examination, as was successful in the case of appendicitis [62]. Both prospects align with the shift towards virtual health implementation formats in light of the health system strain imposed COVID-19 pandemic.

### Potential clinical applications:

These preliminary acceptability and feasibility results suggest that the Callascope self-exam technique could be integrated into existing women’s health services at the primary care level in Peru. While many studies have emphasized the importance of effective, low-cost, primary prevention strategies such as HPV self-sampling [63], few have examined methods for the triage of HPV screen-positive women. There is a critical need for point innovations to correctly identify positive women who need follow-up care. Visual Inspection with Acetic acid (VIA) has been used as a simple, inexpensive test where trained providers can use a speculum, flashlight, and acetic acid to identify early cervical lesions through aceto-whitening of abnormal cells [64,65]. One study [65] compared VIA, cytology, and colposcopy for triage of a large sample of underserved women in Hyderabad, India. Although cytology performed substantially better than VIA in terms of sensitivity, the cost and feasibility of cytology in resource-limited settings was prohibitive. Researchers concluded that VIA was the optimal triage technique for these settings in terms of both sensitivity and cost-effectiveness. After the necessary design modifications have been integrated and validated, home-or clinic-based Callascope-enabled cervix imaging posits an alternative triage strategy to VIA for the triage of HPV positive women. Outfitting the Callascope with functional mechanisms for autonomous application of acetic acid (for the VIA) is critical to this endeavour and could be in the hands of the women but monitored remotely by a health provider.

### Integration into primary prevention programs:

Study participants were community health volunteers of the HOPE program, a social enterprise that recruits and trains community women to sell the entire HPV testing service from self-sampling kit to laboratory to receipt of results to their neighbours and peers at a fraction of the commercial cost. To date, the program website reports that 6,000+ tests have been distributed and 800+ screen-positive women identified [66]. However, primary prevention services in the community are still lacking for women who receive a positive test result. Callascope cervical imaging paired with a decision-making algorithm could bridge this gap by providing another simple, effective tool for triage of HPV positive women. Task shifting the point of diagnosis from highly trained providers to the women themselves, or even just to the nurses or midwives, could strengthen primary screening programs. Studies indicate that task shifting has the potential to address the insufficient health workforce, and reduce costs without comprising on health outcomes, thereby improving the health delivery system, but only with the provision of adequate training and supervision [67,68].

### Addressing social barriers:

In addition to mitigating structural barriers that prevent women from accessing and utilizing cervical cancer screening services, the Callascope has the potential to directly address key social barriers. The standard clinic-based pelvic exam format used to screen for cervical cancer is considered a source of anxiety and fear for many women, specifically the use of the “uncomfortable” and “painful” duckbill speculum [69,70]. Despite this well-studied fact, the speculum is still a necessary part of the modern gynecologic exam. Another reason cited for women avoiding the pelvic exam is the vulnerable position and exposure of their reproductive parts during the exam which provokes embarrassment and humiliation apart from the physical discomfort created by the duckbill speculum [71,72]. The ability to complete a self-examination with the Callascope could address these barriers three-fold 1) by removing the need for the speculum, 2) by increasing knowledge and awareness of the reproductive anatomy through the process of self-exploration and visualization of the anatomy or ‘active learning,’ which in turn could address feelings of shame and stigma associated with reproductive health services [73], and 3) by allowing women to privately and autonomously complete another step of the screening process (for example for women who are already HPV positive after a self-sampling) without being subjected to the standard pelvic exam format.

### Other applications:

Mistrust in the Peruvian healthcare system has been well-documented [74], and some communities are still recovering from government-funded forced sterilization programs carried out against indigenous women living in the Andean region (1996-2000) [75] engaging women in their reproductive healthcare through an interactive exam format could strengthen community and health system relationships between provider and patient. The process of community engagement has made a measurable difference in the success of other prevention programs in Peru such as TATI, where teams reported changes in the equity of relationships with stakeholders [76]. Studies have shown that incorporating advocacy and civil society groups as part of public health interventions to give space for communities of survivors to come together to educate their peers, dispel myths, and advocate for policy changes, can greatly improve follow-up rates [77]. Further, the focus group demonstrated that participants found the self-exam experience to be insightful, educational, and empowering. Educational pedagogical universally shows that hands-on, interactive, and experiential learning improves performance and participation rates [78]. Thus, the Callascope self-exam could have utility as an educational device targeted at teens and young adults.

### Other findings:

One unanticipated outcome of this study was concerns raised by the presence of vaginal/cervical fluids or “discharge”. Participants were not aware that some fluid is normal, and many had specific concerns regarding the volume and consistency of the fluid. First and foremost, the virtual training package should include sufficient resources to prepare women to see vaginal/cervical fluid and information to connect them with local providers should their concerns persist. Unfortunately, availability of low-cost, effective, and simple-to-use Point-Of-Care (POC) diagnostic tests for Sexually Transmitted Infections (STI) is woefully lacking and the development and validation of innovative STI diagnostic technologies has been identified as a top priority by the World Health Organization [79,80]. Regardless, the Callascope intervention package should include adequate educational information and resources for follow-up evaluation related to STIs or reproductive tract infections (as bacterial vaginosis).

### Limitations and future directions:

It is important to recognize that the self-examination and cervix imaging technique disrupts the status quo by shifting a critical primary cervical cancer prevention step from the providers to the patients themselves. In the past, disruptive innovations that displaced ingrained labor practices have been met with resistance. The now ubiquitous breast cancer self-examination, promoted by the WHO as an important awareness and first-line of defense technique, was introduced in the 1950s [81]. However, resistance from healthcare providers delayed its integration into international guidelines until the 1990s [81]. As such, future studies should assess not only improvements made to the device prototype, but also provider acceptability of both the Callascope medical device and also the practice of woman self-imaging their own cervix. An expansive policy analysis should be completely concurrently to identify regulatory considerations that could prevent sustaining and scaling the self-imaging model long-term, such as provider payment, medical device safety clearance, and non-health sector factors. Notably, the study population was already sensitized to reproductive health and cervical cancer at the time of enrolment through their participation in the HOPE HPV Program as community health workers. Thus, acceptability should be assessed in a larger sample size and among a more representative study population.

Further, the study was completed by women autonomously outside of a clinic-setting (at-home) with no medical, professional, or health worker supervision and relatively minimal training. Another Callascope pilot study conducted in 2018 in Durham, NC, United States was designed differently. Women completed two exams. The first was supervised in a clinic. A nurse was on call to help at any time during the examination process, and she evaluated the image for quality and accuracy before giving the woman a self-exam kit to perform a second examination autonomously from her home. Notably, each participant had 7 full days to complete the exam and return the device, whereas in this study participants had 72 hours. This could have been one factor owing to the significantly better image quality obtained in the US cohort. In 2019 another pilot study was conducted in Accra, Ghana. This cohort of women completed one self-exam in a clinic-setting, but the self-exam was guided by a nurse who told the patient when the cervix was in focus and cantered, again leading to higher quality of images. These lessons are considered for future effectiveness and implementation studies in Peru. For example, provider-guided Callascope self-imaging could be possible through telemedicine capabilities, relevant in the context of COVID-19 and the increasing push towards virtualization of the screening process.

## Conclusion

This pilot acceptability and feasibility study has demonstrated that women community health volunteers in Peru are both willing and able to complete a self-exam to image their own cervix. They have also suggested improvements to the Callascope prototype-including lengthening the handle, overlaying the asymmetrical tip with silicon, optimizing the manual focus and resolution. Essential to device and intervention success will be redesigning the self-exam kit to include a robust virtual instruction manual that can be downloaded as a software application for free, including numerous representative videos and images to inform women’s expectations about normal variations in mucus, shape and orientation of the cervix, educational resources on STIs information, as well as information on available local services. Overall, task shifting cervical cancer screening to women themselves could mitigate several structural barriers preventing compliance and utilization of primary prevention services, addressing key social barriers directly. Health behavior research suggests that the process of visualizing your own anatomy has utility also as an educational tool. HPV and HIV self-testing research suggests that autonomous testing formats, like the Callascope self-exam, can increase participation and follow-up rates. However, it is critical to evaluate provider acceptability, perform effectiveness studies and policy analysis to identify potential regulatory barriers to task-shifting upstream into non-clinic settings.

## Figures and Tables

**Table 1:** Participant demographics, prior experience with pelvic exams and perception of ease of use of Callascope assessed with pre-exam survey.

Participant demographics		
Age (years) Median (range)	45 (31-59 yrs)	
Body Mass index (BMI) Median (range)	30(22.7-34.8)	
	Normal	1(7%)
	Overweight	6(40%)
	Obese	8(53%)
Marital status	Single/widowed	3(20%)
	married/partner	12(80%)
Education level	Primary or less	0
	High school	7(47%)
	Technical	7(47%)
	University	1(7%)
Months of participation in HOPE	0 to 5	5(33%)
	6 to 11	3(20%)
	12+	7(47%)
Vaginal births	0	2(14%)
	44228	8(53%)
	44319	5(33%)
Number of prior speculum exams	1 to 5	3(20%)
	6 to 10	9(60%)
	>10	3(20%)
Experience with pain during prior speculum exams	No pain	3(20%)
	Slight pain	4(27%)
	Moderate or extreme pain	8(47%)
Perception of Callascope ease-of-use from appearance	Easy	5(33%)
	Hard	10(67%)
	Very hard	0

**Table 2:** User experience with Callascope self-exam (post-exam survey).

User experience with Callascope self-exam		
Ease of following Callascope instructions	Very easy/easy	14(93%)
	Hard	1(7%)
Number of calla inserter used by participant	Very hard	0
	1 (small)	8(54%)
	2 (large)	3(20%)
	3 (tried both)	4(27%)
Reported time to complete the exam with the Callascope (minutes)	1-3	10(67%)
	4-6	4(27%)
	>6	1(7%)
Ease of use the Callascope to visualize the cervix	Very easy/easy	7(47%)
	Hard	8(53%)
	Very hard	0(0%)
Discomfort level of Callascope use	None	7(47%)
	Slight	5 (33%)
	Moderate	3 (20%)
Pain level of Callascope moving to visualize the cervix	None	12(80%)
	Slight	2(13%)
	Moderate	1(7%)
Comfort of using Callascope compared with speculum	Much worse/worse	0
	Better	8(53.3%)
	Much better	7(46.7%)
Overall experience using Callascope compared with speculum	Much worse/worse	0
	Better	7(46.7%)
	Much better	8(53.3%)

**Table 3:** Association of ease of use of Callascope, discomfort, pain and quality of photos with Body Mass Index (BMI) and number of reported vaginal births.

	BMI		Number of vaginal births	
	R_s (Rho)_	p-value	R_s (Rho)_	p-value
Ease of use the Callascope to visualize the cervix	0.5	0.038*	0.06	0.692
Discomfort level of Callascope use	0.27	0.348	0.3	0.069
Pain level of Callascope moving to visualize the cervix	0.45	0.021*	0.03	0.289
Photo quality of at least one image	0.12	0.53	0.42	0.135

**Table 4:** User experiences, summary of emergent themes from reflection and focus group.

Summary of emergent themes from reflection and focus group, user experiences		
Theme	Description	Representative quotes
Initial perception of the device and the process of self-examination	Many women indicated fear before starting exam, attributed to device texture and/or concept of self-imaging their own cervix.	“Doing my own exam sounded a bit complicated at the beginning.” “It really made me sweat.”
		“My first impression, I thought it was going to graze [my vaginal walls], it was going to hurt because of how rough it felt when I first saw it.”
Exam preferences	Some women preferred to use the Callascope privately, but many women completed the exam with assistance or guidance from household members.	“I waited for everyone to leave for work, locked my door and started to insert.”
		“When my husband was sleeping and I started, though I was a little bit nervous.”
		“During all this my husband woke up and he also saw the image. He was looking at the cell phone while I was moving it. He would say, move it a little more, a little more up. He took my picture, and I would wait [for him to] see.”
Importance of following the instructions and having some support	Following instructions were mentioned by several women as an important part of their experience.	“When introducing it, following all the steps is important”, because I think that if I had not had that practice-seeing and doing it-I may not have done it [on my own].”
		“At that moment, I called Dr. Maria asking what I can do to see my uterus, yeah. [She] told me to do it like this and like that.”
		“When I got home I followed all the instructions.”
Comfort of exam	Most women indicated no discomfort during the self-examination.	“I have done it without pain, without discomfort, without anything, all calm.”
		“I didn’t feel pain.”
		“I have not felt much discomfort.”
		“Everything went fine; I did not feel any pain.”
Better experience than with speculum and provider	Some women specifically stated that the experience was much better (comfort, fear, etc.) than a speculum-based pelvic exam.	“Seeing it like that without pain, without [feeling] nothing, because I’ve had very bad experience[s] with the speculum in the hospitals where I’ve had them [exams] done with, um, pain or burning even on the day after too, uncomfortable. But now not with the Calla; I have felt very good and didn’t feel anything.”
How the whole experience of using the callascope was seen	All women indicated that some aspect of the experience was positive and insightful.	“I was so interested about what this was, the cervix, to see it in this way.”
		“For me it has been a very pleasant experience, because it is the first time that I can see [this] part of my body, my organism through a camera.”
		“It has been a really great experience, something new.”
		“For me, it was an experience, a very good one to be honest.”
		“It was a good experience and above all, it allows us to confirm how our cervix is doing. ”
		“I really would also recommend other people who can, to have it done, because it is not the same as doing it yourself as when a doctor does it, a professional. ”
		“What struck me was seeing how the body is, always being thorough to see on both sides to visualize well with the camera, because when you have an exam, uh, with a, well in the health area, they do it as if separate from you.”
Would recommend use or try again	All women indicated that they would likely recommend device use or try again.	“The experience was really very favorable; simple without pain and I would recommend women do it, I really do. That is my experience.”
		“I would really recommend other women to do it because it is important for us to do it.”

**Table 5:** Challenge encountered during self-examination, summary of emergent themes from reflection and focus group.

Summary of emergent themes from reflection and focus group, challenges encountered during self-examination		
Theme	Description	Representative quotes
Discharge or blood obstructed self-examination	Most women found vaginal discharge or blood during their exam, for many this obstructed their ability to visualize the cervix.	“I saw a little bit of mucus or maybe discharge.”
		“I saw my cervix a little more reddish, more like, with a little bit of blood.”
		“I stretched forward and heard a sound and saw a little bit of blood. I got scared, and wondered what may have happened, but there was no pain.”
		“[There was] a little pain because I was having discharge.”
		“And saw that I had enough fluid, it was like my cervix was swimming in fluid, but it was a white fluid and from moving, it moved it. It was foamy and it was so interesting to me that I kept moving it,”
Fear of unknown discharge during exam	Vaginal discharge scared many of the women; they were unsure what they were seeing and questioned whether or not they were healthy.	“I got a little scared because I saw this, something white.”
		“I was scared because I saw something, discharge.”
		“I hope its fine, even with something’s, I think, you can see a lot of discharge.”
		“I had enough fluid as if my uterus was dancing in fluid.”
		“My fear was not being able to correctly identify how my uterus was because I saw something like, in the center kind of yellowish stain”
Difficulty finding cervix	Most women indicated difficulty in finding what they thought appeared to be the cervix.	“I really do not know if I did the test well”
		“My experience [finding the cervix] was a little difficult at the beginning.”
		“The only thing that shocked me was seeing the photo, seeing and trying to plan, trying finding and seeing the center the cervix, to not be able to do it.”
		“I did not see the cervix well so I used calla 2.”
